# The acute transcriptome response of the midbrain/diencephalon to injury in the adult mummichog (*Fundulus heteroclitus*)

**DOI:** 10.1186/s13041-019-0542-4

**Published:** 2019-12-30

**Authors:** Eleanor C. Bisese, Chandler M. Ciuba, Amelia L. Davidson, Akanksha Kaushik, Sabrina M. Mullen, Jeremy L. Barth, E. Starr Hazard, Robert C. Wilson, Gary Hardiman, David M. Hollis

**Affiliations:** 1 Department of Biology, Furman University, 3300 Poinsett Highway, Greenville, SC 29613 USA; 2 Department of Regenerative Medicine & Cell Biology, Medical University of South Carolina, 171 Ashley Avenue, Charleston, SC 29425 USA; 3 Computational Biology Resource Center, Medical University of South Carolina, 171 Ashley Avenue, Charleston, SC 29425 USA; 4 Pathology and Laboratory Medicine, Medical University of South Carolina, 171 Ashley Avenue, Charleston, SC 29425 USA; 5 Department of Medicine, Medical University of South Carolina, 171 Ashley Avenue, Charleston, SC 29425 USA; 6 School of Biological Sciences & Institute for Global Food Security, Queen’s University Belfast, Belfast, Northern Ireland BT9 5DL UK

**Keywords:** Brain injury, Acute, Mummichog, Transcriptome, Midbrain, Diencephalon, *pim-2*-like, *syndecan-4*-like, *cd83*

## Abstract

Adult fish produce new cells throughout their central nervous system during the course of their lives and maintain a tremendous capacity to repair damaged neural tissue. Much of the focus on understanding brain repair and regeneration in adult fish has been directed at regions of the brainstem and forebrain; however, the mesencephalon (midbrain) and diencephalon have received little attention. We sought to examine differential gene expression in the midbrain/diencephalon in response to injury in the adult fish using RNA-seq. Using the mummichog (*Fundulus heteroclitus*), we administered a mechanical lesion to the midbrain/diencephalon and examined differentially expressed genes (DEGs) at an acute recovery time of 1 h post-injury. Comparisons of whole transcriptomes derived from isolated RNA of intact and injured midbrain/diencephalic tissue identified 404 DEGs with the vast majority being upregulated. Using qPCR, we validated the upregulation of DEGs *pim-2*-like, *syndecan-4*-like, and *cd83*. Based on genes both familiar and novel regarding the adult brain response to injury, these data provide an extensive molecular profile giving insight into a range of cellular processes involved in the injury response of a brain regenerative-capable vertebrate.

## Background

Adult fish possess tremendous neural regeneration capabilities. While the adult mammalian brain is severely limited in its ability to self-repair, fish exhibit a tremendous capacity for neural regeneration and thus, a remarkable ability to recover from brain injury as they readily replace damaged cells due to their ability to constitutively proliferate new cells [1]. Thus, due to their having exceptional potential to regenerate neuronal tissue post-injury, fish are an excellent model for adult brain regeneration-competency [2]. Factors responsible for the differences in adult brain cell proliferation, and subsequent neurogenesis, between species of different vertebrate classes remain largely unknown. Therefore, elucidating the genes involved in the adult fish brain reparative process has the potential to better understand the molecular mechanisms underlying these discrepancies.

Cell proliferation and neurogenesis are abundant throughout the adult fish brain [3]. Much of the focus on adult fish brain repair has been directed at neuronal tissue of the forebrain and brainstem, however, the mesencephalon (midbrain) and diencephalon have received relatively little experimental study with regard to reparative neurogenesis relative to the other regions [4]. Studies using genomic approaches, specifically RNA-seq, to assay the molecular signals associated with brain regeneration and neurogenesis in the fish brain are beginning to uncover significant genes and pathways [5]. To further this end, we used RNA-seq to examine the acute response of the midbrain/diencephalon of the mummichog (*Fundulus heteroclitus*). While the zebrafish (*Danio rerio*) has been widely used to examine molecular mechanisms of pathological conditions such as brain repair, alternative fish species, such as the mummichog, allow for potential novel gene function discovery due to differential subfunctionalization of genes between the different fish lineages [6].

## Materials and methods

A detailed description of all experimental methods including animal care, surgical process, Nissl staining, RNA-seq, and qPCR can be found in Additional file 1. A mechanical lesion was administered to the midbrain and underlying hypothalamic diencephalon of anesthetized adult mummichogs. After injury, fish were given an acute recovery time of 1 h prior to sacrifice. To ensure lesion accuracy, Nissl stain was performed on 20 μm thick frontal sections of the midbrain/diencephalon to visualize the injury using light microscopy.

For RNA-seq, the total RNA was isolated from the lesioned side of the midbrain/diencephalon as well from the contralateral, intact side of the midbrain/diencephalon of ten fish. The total RNA from each sample was used to prepare RNA-seq libraries which were clustered at concentrations to ensure at least 50 million reads per sample. Differential gene expression was inferred using DESeq2 [7]. Transcript counts from DESeq2 analysis were ranked according to adjusted *p*-value (q). The false discovery rate (FDR) was set at both q ≤ 0.1 or < 0.4 on the premise that the lower threshold was appropriately very stringent for identifying high-probability DEGs, while the higher threshold was more permissive and thus, avoided the loss of data via false negatives and would therefore aid discovery in the downstream functional analyses by providing a larger input gene list as we have demonstrated previously [8, 9]. The gene set identified by q ≤ 0.4 was then analyzed with the ToppFun tool [10] to find enriched GO terms and pathways. The systems level output (GO and Pathway) was itself subjected to FDR testing thereby adding rigor to the data analysis [see 8, 9].

To validate DEGs, qPCR was performed (on eight to ten fish distinct from the RNA-seq assay) utilizing gene-specific primers (see Additional file 1: Table S1). Primers were designed for three DEGs of novel functional interest regarding the fish brain response to injury that showed high differential expression**,** which included *pim-2*-like, *syndecan-4*-like and *cd83 molecule* (*cd83*). In addition, for qPCR quality control, primers were designed for a gene of functional interest, *igf-1* (*insulin-like growth factor 1*), to validate its lack of differential expression. Gene expression was normalized to *elongation factor 1 alpha* (*ef1a*) using primers designed in previous work [11]. Further, to determine if a whole midbrain/diencephalon gene expression response was elicited, midbrain/diencephalic tissue from ten fish receiving no injury was also included.

## Results

**Fig. 1:**
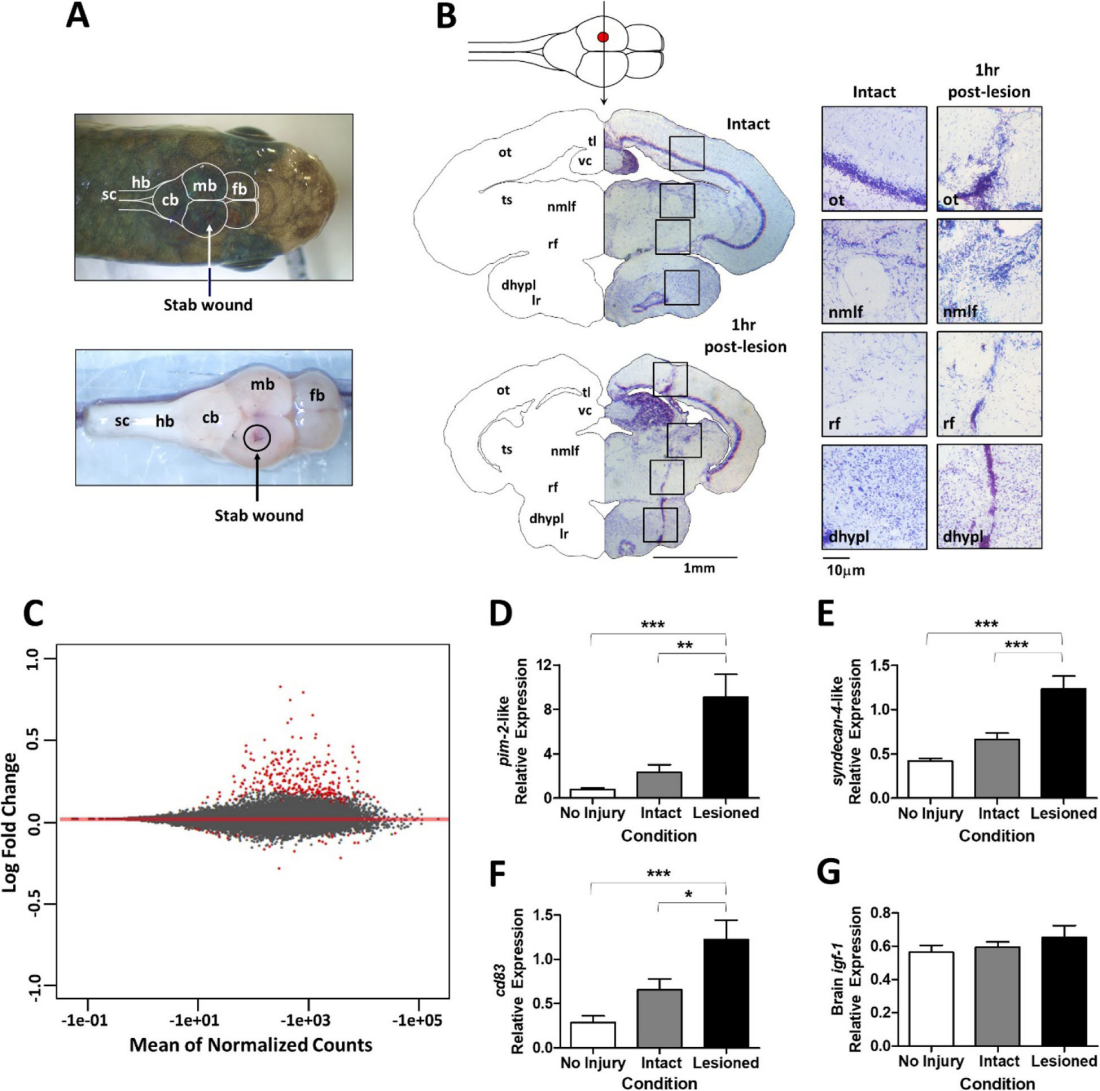
Differentially gene expression in the mummichog midbrain/diencephalon in response to injury at 1 h post-lesion. **a** Placement of the mechanical lesion to the mummichog midbrain/diencephalon. Abbreviations. cb = cerebellum, fb = forebrain, hb = hindbrain, mb = midbrain, sc = spinal cord. **b** Frontal sections of mummichog midbrain/diencephalon with Nissl stain (right; magnification = 25X) and neuroanatomical areas in a mirror image schematic (left). Boxes show regional intact (left column) vs lesioned (right column) tissue (magnification = 160X). Abbreviations: dhypl = diffuse nucleus of the inferior hypothalamic lobe, lr = lateral recess, nmlf = nucleus of the medial longitudinal fascicle, nr = nuclear ruber, ot = optic tectum, tl = torus longitudinalis, ts = torus semicircularis, vc = valvula cerebelli. **c** MA plot of the distribution of expressed genes at 1 h post-injury as determined by RNA-seq. Red dots indicate DEGs with an FDR of q ≤ 0.1 (181 genes) or ≤ 0.4 (223 genes). **d**, **e**, **f** Upregulation of DEGs, *pim-2*-like (q = 5.09E-46) (d), *syndecan-4*-like (q = 5.22E-30) (e), & *cd83* (q = 2.97E-23) (f) at 1 h post-lesion (One-Way ANOVA; *p* < 0.001 for each. Tukey’s Post Test; *** = *p* < 0.0001, ** = p < 0.001, * = *p* < 0.05. *n* = 10 for each). **g** Validation of a non-differentially expressed gene, brain *igf-1* (*n* = 8)

Due to the relatively flattened head of the mummichog, it was ideal for the dorsal application of a mechanical lesion to the midbrain/diencephalon (Fig. 1a). Nissl stain confirmed that the lesion traversed the midbrain optic tectum and tegmental regions and into the underlying hypothalamic tissue of the diencephalon (Fig. 1b). Genes differentially expressed between the lesioned and contralateral intact midbrain/diencephalon tissue were identified using FDR-adjusted *p*-value cutoffs of q > 0.1 and 0.4, respectively. In total, 404 DEGs were identified as differentially expressed with 181 DEGs meeting the FDR cutoff of q ≤ 0.1 (Additional file 2: Table S2). Most differential expression indicated gene upregulation (Fig. 1c), which accounted for nearly 80% of all DEGs and of those with FDRs of q ≤ 0.1, 90% were upregulated. Functional annotation of genes by GO analysis [see 10] revealed a broad range of biological processes indicating a strong influence over cell death and differentiation (see Additional file 2: Table S3).

From the qPCR studies (Additional file 2: Table S4), we validated the differential expression of two genes not previously identified in the fish brain response to injury, which included *pim-2-*like (Fig. 1d) and *syndecan-4*-like (Fig. 1e), both of which are associated with cell proliferation [12, 13]. Also validated was *cd83* (Fig. 1f), which is associated as a regulator of activation in immune cells [14]. However, *cd83* was recently found in neuronal cells, including high expression levels in both the midbrain and hypothalamus indicating possible distinct neural function [15]. Finally, the lack of *igf-1* differential expression was also validated (Fig. 1g). The use of the mummichog midbrain/diencephalon as a novel model for the brain response to injury demonstrates its potential to identify genes yet to be discovered with putative roles in the brain reparative processes in regenerative-capable vertebrates.

## Supplementary information

**Additional file 1 Table S1.** List of qPCR primers used to validate specific RNA-seq data. With the exception of the *ef1a* primers, all primers were designed using the online primer design tools from Integrated DNA Technologies (Coralville, IA) and commercially synthesized by the company. Primers for *ef1a* based on **[**see 1].

**Additional file 2 Table S2.** List of differentially expressed genes between control intact midbrain/diencephalic tissue and lesioned midbrain/diencephalic tissue at 1 h post-injury using DESeq2. The list contains significantly expressed genes (q < 0.4) and was sorted by fold-change using log2(FC) of down-regulated and up-regulated transcripts. The Gene IDs of differentially expressed genes *pim-2*-like, *syndecan-4*-like, and *cd83* used in qPCR validation assays are shown in bold text. A solid line delineates the first 181 genes with FDRs < 0.1 from the following 223 genes with FDRs < 0.4 that did not make the 0.1 cutoff. **Table S3.** Functional annotation biological processes of DEGs identified by RNA-seq. Gene Ontology (GO) enrichment analysis was performed by ToppFun web server (https://toppgene.cchmc.org/enrichment.jsp). Differentially expressed genes (q < 0.4) from DESeq2 analysis were entered into ToppGene. ToppFun selected analogous human symbols (e.g. cd83 became CD83) for about half of the 404 DEGs entered. The table is the Biological Processes portion. **Table S4.** Cycle threshold (Ct) values from qPCR. Each value represents the average value from reactions performed in triplicate. Housekeeper gene Ct scores were retested when reagents for an assay were obtained from different kits. Sample sizes were from eight to ten individuals.

## Data Availability

The RNA-seq data sets generated were deposited and are available at the NCBI Sequencing Read Archive database (Accession: GSE137451).

## Abbreviations

cd83: Cluster of differentiation 83
DEGs: Differentially expressed genes
ef1a: Elongation factor 1 alpha
GO: Gene ontology
igf-1: Insulin-like growth factor 1
pim-2: Proviral integration site for moloney murine leukemia virus, isoform 2
q: Adjusted p value
qPCR: Quantitative polymerase chain reaction
RNA-seq: RNA sequencing
