# Supplementary material for: The acute transcriptome response of the midbrain/diencephalon to injury in the adult mummichog (Fundulus heteroclitus)
Source: Mol Brain. 2019 Dec 30;12:119. doi: 10.1186/s13041-019-0542-4 (PMC6937918; doi:10.1186/s13041-019-0542-4)
Supplement: Supplementary file 1 — Additional file 1 Table S1. List of qPCR primers used to validate specific RNA-seq data. With the exception of the ef1a primers, all primers were designed using the online primer design tools from Integrated DNA Technologies (Coralville, IA) and commercially synthesized by the company. Primers for ef1a based on [see 1]. [file 13041_2019_542_MOESM1_ESM.docx]

**The acute transcriptome response of the midbrain/diencephalon to injury in the adult mummichog (*Fundulus heteroclitus*)**

**Eleanor C. Bisese^1^, Chandler M. Ciuba^1^, Amelia L. Davidson^1^, Akanksha Kaushik^1^,**

**Sabrina M. Mullen^1^, Jeremy L. Barth^2a^, E. Starr Hazard^2b^, Robert C. Wilson^2c^,**

**Gary Hardiman^2d, 3^ and David M. Hollis^1^**^*^

^1^Department of Biology, Furman University, 3300 Poinsett Highway, Greenville, SC 29613

^2^Medical University of South Carolina, 171 Ashley Avenue, Charleston, SC 29425

^2a^Department of Regenerative Medicine & Cell Biology

^2b^Computational Biology Resource Center

^2c^Pathology and Laboratory Medicine

^2d^Department of Medicine

^3^School of Biological Sciences & Institute for Global Food Security, Queen’s University Belfast,

Belfast, BT9 5DL, Northern Ireland, UK

**Corresponding author:**

David M. Hollis, PhD

Furman University

Department of Biology

3300 Poinsett Highway

Greenville, SC 29613

Tel: 864-294-2306

Fax: 864-294-2058

Eleanor C. Bisese, email: [ellie.bisese@furman.edu](mailto:ellie.bisese@furman.edu)

Chandler M. Ciuba, email: [chandler.ciuba@furman.edu](mailto:chandler.ciuba@furman.edu)

Amelia L. Davidson, email: [amelia.davidson@furman.edu](mailto:amelia.davidson@furman.edu)

Akanksha Kaushik, email: [akanksha.kaushik@furman.edu](mailto:akanksha.kaushik@furman.edu)

Sabrina M. Mullen, email: [sabrina.mullen@furman.edu](mailto:sabrina.mullen@furman.edu)

Jeremy L. Barth, email: [barthj@musc.edu](mailto:barthj@musc.edu)

E. Starr Hazard, email: [hazards@musc.edu](mailto:hazards@musc.edu)

Robert C. Wilson, email: [wilsorc@musc.edu](mailto:wilsorc@musc.edu)

Gary Hardiman, email: [g.hardiman@qub.ac.uk](mailto:g.hardiman@qub.ac.uk)

*David M, Hollis, e-mail: [david.hollis@furman.edu](mailto:david.hollis@furman.edu)

**This file includes:**

Materials and Methods

Table S1

**Materials and Methods**

**Animals**

The Furman University Institutional Animal Care and Use Committee (IACUC) approved the use of animals or their tissues in the study (IACUC protocol # 01-19-06). Adult mummichogs were obtained from Aquatic Research Organisms (Hampton, NH). Daily care for the mummichogs was based on [1]. Mummichogs were housed in glass aquarium tanks at room temperature (22^o^C) each containing 40L of dechlorinated water at 10ppt marine salt (Instant Ocean®) on a 12:12 light/dark cycle. Fish were fed daily with fish flakes and blood worms (Tetra®).

**Mechanical Lesion**

The surgical process used was following the methods of [2], with modification for injury to the midbrain/diencephalon. Fish were anesthetized with 2-phenoxyethanol diluted in tank water (1:500). Fish remained in the anesthetic until they could no longer move or right themselves (≈ 5min). They were then placed in a paper towel soaked with the 2-phenoxyethanol diluted in tank water. Under dissecting microscope, fish were held upright for direct illumination of the head, which allowed visualization of the pigmented meninges overlying the tectal hemispheres of the dorsal midbrain. A sterile 23G syringe needle was placed directly over one tectal hemisphere of the midbrain and slowly rotated back and forth while gently pushing down. This needle was inserted to the depth of its bevel (≈ 2mm) which allowed the lesion to penetrate the optic tectum, tegmental regions of the midbrain and underlying hypothalamic tissue of the diencephalon. After injury, fish were placed in tank water (10ppt saline) for recovery and were swimming under their own power within 5min. They were then placed into a separate tank until sacrifice, which occurred 1hr post-lesion.

**Nissl Stain**

For sacrifice, mummichogs were placed in tank water containing 2-phenoxyethanol (1:1000). Fish remained in the anesthetic until they could no longer move or right themselves

(≈ 2min). Fish were sacrificed by rapid decapitation. Brains were removed and fixed in 4% paraformaldehyde (in 0.1M phosphate buffer: PB) for 1hr. After fixing, the brains were cryoprotected in 30% sucrose (in 0.1M PB) overnight. After cryoprotection, brains were embedded in Histoprep Frozen Tissue Embedding Medium (ThermoFisher Scientific), and stored in -80^o^C until sectioning. Brains were cut in frontal section on a cryostat at 30^o^C at a thickness of 20μm and briefly (5s) thaw-mounted at 35^o^C on Superfrost Plus1 positive charged microscope slides (Shandon, Inc.) before being stored at -80^o^C until use. For staining, the tissue was removed from -80^o^C and allowed to warm to room temperature (22-23^o^C) for 5min. The tissue was then further baked on the slides for 5min at 35^o^C for 4min and then removed and allowed to cool back to room temperature for 1min. The tissue was then fixed in 4% paraformaldehyde (in 0.1M PB) for 30min, washed in 0.1M PB (5min), followed by 95% ethanol (15min), 70% ethanol (1min) and 50% ethanol (1min), 0.1M PB (2min), 0.1M PB (1min), and then stained in cresyl violet (15min). Excess stain was removed by dipping the tissue in distilled H_2_O, 95% ethanol, and 100% ethanol. The tissue was then cleared in SafeClear (15min) and cover-slipped in Permount. The tissue sections were observed using a Nikon OptiPhot light microscope and microphotographs captured with a Jenoptik ProgResR Gryphax Subra HD USB3 digital camera (Jena, Germany). The microphotographs were analyzed using GRYPHAX V1.0.2.50 software (Jenoptik Optical Systems; Jena, Germany). Nomenclature used to refer to neuroanatomical regions was based on previous work [3-8].

**Tissue Collection and RNA Isolation**

For sacrifice, mummichogs were placed in tank water containing 2-phenoxyethanol (1:1000). Fish remained in the anesthetic until they could no longer move or right themselves (≈ 2min). Fish were sacrificed by rapid decapitation and midbrain/diencephalic tissue was isolated, and the left and right hemispheres were separated, weighed, and immediately homogenized in QIAzol® Reagent (QIAGEN) for total RNA isolation using the QIAzol® RNeasy Lipid Tissue Mini Kit (QIAGEN) along with the RNase-Free DNase Set (QIAGEN) to remove genomic DNA.

For RNA-seq, ten mummichogs were used. From each fish, total RNA was isolated from the lesioned hemisphere of the midbrain/diencephalon (n = 10; five left lesioned, five right lesioned) as well as the contralateral, intact hemispheres (n = 10; five right intact, five left intact). For qPCR validation of differentially expressed genes, the total RNA of ten different mummichogs were similarly isolated. However, as an additional control to determine if a whole midbrain/diencephalon gene expression response was elicited, a further ten animals receiving no injury were also used, where the total RNA of either their left (n= 5) or right (n = 5) midbrain/diencephalon hemisphere was isolated.

**RNA-seq and Functional Enrichment Analysis**

For RNA-seq, total RNA integrity was verified on an Agilent 2200 TapeStation (Agilent Technologies, Palo Alto, CA) utilizing samples with RINs ≥8. Total RNA (100-200 ng) was used to prepare RNA-Seq libraries using the TruSeq RNA Sample Prep Kit following the protocol as described by the manufacturer (Illumina, San Diego, CA). Libraries were clustered at a concentration to ensure at least 50 million reads per sample on the cBot as described by the manufacturer (Illumina, San Diego, CA). Clustered RNA-seq libraries were paired-end sequenced using version 4 with 1X50 cycles on an Illumina HiSeq2500. Demultiplexing was performed utilizing bcl2fastq-1.8.4 to generate Fastq files. The samples averaged ~63 million reads with Phil’s Read Editor (PHRED) quality scores comfortably greater than 30 [9]. Sample read files were then processed to trim adapter sequences with the program CutAdapt [10]. We then downloaded the current *Fundulus heteroclitus* genome from NCBI (GCF_000826765.1_Fundulus_heteroclitus-3.0.2_genomic). We created a local *Fundulus* STAR (Spliced Transcripts Alignment to a Reference) index and aligned the trimmed sample read files via the STAR aligner (https://www.ncbi.nlm.nih.gov/pubmed/23104886). This gave a uniquely mapped reads rate of ~93% and an average ~58 million reads per sample. STAR output bam files were counted on a per gene level by the DESeq2 python utility htseq_counts.py (<https://academic.oup.com/bioinformatics/article/31/2/166/2366196>) that outputted gene names (e.g. gene1513) and raw counts per gene.

To infer differential gene expression with robust statistical power, we utilized DESeq2 to test for differential expression based on a model using negative binomial distribution [11]. The DESeq2 script accounted for the experimental effect as well as the replicate design. Transcript count data from DESeq2 analysis was ranked according to adjusted p-value. Our methodology followed the premise that transcripts were sorted according to their q-value, which is the smallest false discovery rate (FDR) at which the transcript was called significant where the FDR was the expected fraction of false positive tests among significant tests. We then searched for groups of genes that collectively enriched GO terms or pathways. In many cases these were present at an FDR of 0.4, but may not have been present with a more stringent cutoff ≤ 0.1, in which case meaningful biological data would have been lost. Furthermore, the systems level analysis we performed (GO and Pathway) was itself subjected to FDR testing thereby adding rigor to the data [12, 13]. We reported 181 DEGs at FDR < 0.1 and 404 DEGs at FDR < 0.4. Gene symbols and geneid numbers were extracted from the NCBI gene_info (ftp://ftp.ncbi.nlm.nih.gov/gene/DATA/) archive with a series of custom PERL and BASH scripts. This information was then merged with the Gene-based DESeq2 analysis.

**Primers**

Gene-specific primers used for qPCR were designed from the mummichog gene sequences for *pim-2*-like (Accession# XM_012855954), *syndecan-4*-like (Accession # XM_012853907), *cd83* (Accession # XM_012856051), and brain *igf-1* (Accession # XM_012873804.1). All isolated fragments of each gene were within their open reading frames. Gene expression was normalized using a 55bp fragment of the *elongation factor 1 alpha* (*ef1α*) using gene-specific primers based on previous work [14]. Primer specificity was verified, and optimal annealing temperatures identified, using endpoint gradient PCR followed by gel electrophoresis to ensure that only a single band of predicted size from the within the open reading frame (genomic DNA was removed during RNA isolation) occurred for each primer set. Additionally, melt curve analysis was performed with each qPCR assay to further ensure primer specificity. Primer sequences are shown on Table S1.

**qPCR**

The qPCR was carried out following the manufacturer’s instructions for the iTaq Universal SYBR1Green One-Step kit reaction method (BioRad). A total of 36ng of total RNA was used per 12μl reaction (3ng/μl per reaction). All reactions were performed in triplicate. Reverse transcription was carried out using iScript Reverse Transcriptase (BioRad) and the generated cDNA was used as a template for PCR amplification. The cycling conditions were, for the reverse transcription, 50^o^C (10min), which was then followed by qPCR consisting of 95^o^C (1min), then 35 cycles of 95^o^C (15s), and 65^o^C (60^o^C for *ef1α*) (1min). Reverse transcription and qPCR amplification were performed on an Eppendorf Mastercycler1 ep realplex2 thermal cycler. Standard curves used to compute expression values for *ef1α*, *pim-2*-like, *syndecan-4*-like, *cd83* and brain *igf-1* were generated from total RNA of mummichog brain tissue with individual 12μl reactions, performed in triplicate, ranging from 1.6ng to 1000ng in total RNA (0.13 ng/μl to 83.33 ng/μl). Statistical significance for differences in relative expression levels between the three conditions (no injury, contralateral intact, and lesioned) was evaluated by One-Way ANOVA (P < 0.05) followed with Tukey’s post-test (P < 0.05). Statistics were performed using GraphPad Prism (5.0) software.

**Table S1.** List of qPCR primers used to validate specific RNA-seq data. With the exception of the *ef1a1* primers, all primers were designed using the online primer design tools from Integrated DNA Technologies (Coralville, IA) and commercially synthesized by the company. Primers for *ef1a1* based on **[**see 1].

| **Gene Name** | **Primer Sequence (5’ → 3’)** | **Tm** | **Fragment Size** | **R^2^** |
| --- | --- | --- | --- | --- |
| *pim-2-like* | Fwd: CAGCGGCTCTCGGATGGACTC  Rev: CAGTCTGGCCCACTGCTGAACC | 65°C | 75bp | 0.98 |
| *syndecan-4-like* | Fwd: GTGGACGAAGTGGACCTCGTC  Rev: GACCTCCGTGCTGTTGAAGACG | 65°C | 138bp | 0.99 |
| *cd83* | Fwd: CCATGACCTCTTCCTGTCCAACGT  Rev: CAGAGTGAGCAGAACTTTGCCCTC | 65°C | 112bp | 0.99 |
| *igf-1* | Fwd: TGTCTGTGGAGAGAGGGGCT  Rev: AGCTCACAACTCTGGAAGCAGCA | 65°C | 108bp | 0.99 |
| *ef1α* | Fwd: GGGAAAGGGCTCCTTCAAGT  Rev: ACGCTCGGCCTTCAGCTT | 60°C | 55bp | 0.99 |

**References**

# 1. MacLatchy DL, Gormley KL, Ibey REM, Sharp RL, Shaughnessy KS, Courtenay SC, Dubé MG, Van Der Kraak GJ. In: Techniques in Aquatic Toxicology, Ostrander G, editor. A short term mummichog (*Fundulus heteroclitus*) bioassay to assess endocrine responses to hormone-active compounds and mixtures. 2005;2(Ch 4):55-91.

2. Schmidt R, Beil T, Strähle U, Rastegar S. Stab wound injury of the zebrafish adult telencephalon: A method to investigate vertebrate brain neurogenesis and regeneration. J Vis Exp*.* 2014;88:e51543.

3. Billard R, Peter RE. A stereotaxic atlas and technique for nuclei of the diencephalon of rainbow trout (*Salmo gairdneri*). Reprod Nutr Dev. 1982;22(1A):1-25.

4. Castro A, Becerra M, Manso MJ, Anadón R. Distribution and development of calretinin-like immunoreactivity in the telencephalon of the brown trout, *Salmo trutta fario*. J Comp Neurol. 2003;467(2):254-69.

5. Folgueira M, Anadón R, Yáñez J. An experimental study of the connections of the telencephalon in the rainbow trout (*Oncorhynchus mykiss*). I: olfactory bulb and ventral area. J Comp Neurol. 2004a;480(2):180–203.

6. Folgueira M, Anadón R, Yáñez J. Experimental study of the connections of the telencephalon in the rainbow trout (*Oncorhynchus mykiss*). II: dorsal area and preoptic region. J Comp Neurol. 2004b;480(2):204-33.

7. Piñuela C, Baatrup E, Geneser FA. Histochemical distribution of zinc in the brain of the rainbow trout, *Oncorhynchos myciss*. I. The telencephalon. Anat Embryol (Berl). 1992a;185(4):379-88.

8. Piñuela C, Baatrup E, Geneser FA. Histochemical distribution of zinc in the brain of the rainbow trout, *Oncorhynchos myciss*. II. The diencephalon. Anat Embryol (Berl). 1992b;186(3);275-84.

9. Andrews S. FastQC: A quality control tool for high throughput sequence data. 2010.

<http://www.bioinformatics.babraham.ac.uk/projects/fastqc/>. Accessed 6 Oct 2011.

10. Martin M. Cutadapt removes adapter sequences from high-throughput sequencing reads.

EMBnet.journal. 2011;17(1):10-12. doi:http://dx.doi.org/10.14806/ej.17.1.200.

11. Love MI, Huber W, Anders S. 2014. Moderated estimation of fold change and dispersion for RNA-seq data with DEseq2. Genome Biol. 2014;15(12):550.

12. Huff M, da Silveira WA, Carnevali O, Renaud L, Hardiman G. Systems Analysis of

the Liver Transcriptome in Adult Male Zebrafish Exposed to the Plasticizer (2- Ethylhexyl) Phthalate (DEHP). Sci Rep. 2018;8(1):2118. doi: 10.1038/s41598-018-20266-8.

13. Hardiman G, Savage SJ, Hazard ES, Wilson RC, Courtney SM, Smith MT, Hollis BW,

Halbert CH, Gattoni-Celli S. Systems analysis of the prostate transcriptome in African-American men compared with European-American men. Pharmacogenomics. 2016;17(10):1129-43.

14. Scott GR, Richards JG, Forbush B, Isenring P, Schulte PM. 2004. Changes in gene

expression in gills of the euryhaline killifish *Fundulus heteroclitus* after abrupt salinity

transfer. Am J Physiol Cell Physiol. 2004;287(2):C300-9.
