# Supplementary material for: The acute transcriptome response of the midbrain/diencephalon to injury in the adult mummichog (Fundulus heteroclitus)
Source: Mol Brain. 2019 Dec 30;12:119. doi: 10.1186/s13041-019-0542-4 (PMC6937918; doi:10.1186/s13041-019-0542-4)
Supplement: Supplementary file 2 — Additional file 2 Table S2. List of differentially expressed genes between control intact midbrain/diencephalic tissue and lesioned midbrain/diencephalic tissue at 1 h post-injury using DESeq2. The list contains significantly expressed genes (q < 0.4) and was sorted by fold-change using log2(FC) of down-regulated and up-regulated transcripts. The Gene IDs of differentially expressed genes pim-2-like, syndecan-4-like, and cd83 used in qPCR validation assays are shown in bold text. A solid line delineates the first 181 genes with FDRs < 0.1 from the following 223 genes with FDRs < 0.4 that did not make the 0.1 cutoff. Table S3. Functional annotation biological processes of DEGs identified by RNA-seq. Gene Ontology (GO) enrichment analysis was performed by ToppFun web server (https://toppgene.cchmc.org/enrichment.jsp). Differentially expressed genes (q < 0.4) from DESeq2 analysis were entered into ToppGene. ToppFun selected analogous human symbols (e.g. cd83 became CD83) for about half of the 404 DEGs entered. The table is the Biological Processes portion. Table S4. Cycle threshold (Ct) values from qPCR. Each value represents the average value from reactions performed in triplicate. Housekeeper gene Ct scores were retested when reagents for an assay were obtained from different kits. Sample sizes were from eight to ten individuals. [file 13041_2019_542_MOESM2_ESM.docx]

**The acute transcriptome response of the midbrain/diencephalon to injury in the adult mummichog (*Fundulus heteroclitus*)**

**Eleanor C. Bisese^1^, Chandler M. Ciuba^1^, Amelia L. Davidson^1^, Akanksha Kaushik^1^,**

**Sabrina M. Mullen^1^, Jeremy L. Barth^2a^, E. Starr Hazard^2b^, Robert C. Wilson^2c^,**

**Gary Hardiman^2d, 3^ and David M. Hollis^1^**^*^

^1^Department of Biology, Furman University, 3300 Poinsett Highway, Greenville, SC 29613

^2^Medical University of South Carolina, 171 Ashley Avenue, Charleston, SC 29425

^2a^Department of Regenerative Medicine & Cell Biology

^2b^Computational Biology Resource Center

^2c^Pathology and Laboratory Medicine

^2d^Department of Medicine

^3^School of Biological Sciences & Institute for Global Food Security, Queen’s University Belfast,

Belfast, BT9 5DL, Northern Ireland, UK

**Corresponding author:**

David M. Hollis, PhD

Furman University

Department of Biology

3300 Poinsett Highway

Greenville, SC 29613

Tel: 864-294-2306

Fax: 864-294-2058

Eleanor C. Bisese, email: [ellie.bisese@furman.edu](mailto:ellie.bisese@furman.edu)

Chandler M. Ciuba, email: [chandler.ciuba@furman.edu](mailto:chandler.ciuba@furman.edu)

Amelia L. Davidson, email: [amelia.davidson@furman.edu](mailto:amelia.davidson@furman.edu)

Akanksha Kaushik, email: [akanksha.kaushik@furman.edu](mailto:akanksha.kaushik@furman.edu)

Sabrina M. Mullen, email: [sabrina.mullen@furman.edu](mailto:sabrina.mullen@furman.edu)

Jeremy L. Barth, email: [barthj@musc.edu](mailto:barthj@musc.edu)

E. Starr Hazard, email: [hazards@musc.edu](mailto:hazards@musc.edu)

Robert C. Wilson, email: [wilsorc@musc.edu](mailto:wilsorc@musc.edu)

Gary Hardiman, email: [g.hardiman@qub.ac.uk](mailto:g.hardiman@qub.ac.uk)

*David M, Hollis, e-mail: [david.hollis@furman.edu](mailto:david.hollis@furman.edu)

**This file includes:**

Table S2

Table S3

Table S4

**Table S2.** List of differentially expressed genes between control intact midbrain/diencephalic tissue and lesioned midbrain/diencephalic tissue at 1hr post-injury using DESeq2. The list contains significantly expressed genes (q < 0.4) and was sorted by fold-change using log2(FC) of down-regulated and up-regulated transcripts. The Gene IDs of differentially expressed genes *pim-2*-like, *syndecan-4*-like, and *cd83* used in qPCR validation assays are shown in bold text. A solid line delineates the first 181 genes with FDRs < 0.1 from the following 223 genes with FDRs < 0.4 that did not make the 0.1 cutoff.

| **Gene ID** | **Gene Symbol** | **Description** | **Base Mean** | **log2(FC)** | **q** |
| --- | --- | --- | --- | --- | --- |
| 105920369 | LOC105920369 | serine/threonine-protein kinase pim-2-like | 267.621 | 0.809 | 5.09E-46 |
| 105918751 | LOC105918751 | syndecan-4-like | 1032.943 | 0.633 | 5.22E-30 |
| 105928246 | LOC105928246 | proheparin-binding EGF-like growth factor | 417.303 | 0.607 | 7.64E-25 |
| 105920451 | cd83 | CD83 molecule | 362.417 | 0.586 | 2.97E-23 |
| 105920594 | cebpa | CCAAT/enhancer binding protein alpha | 439.594 | 0.503 | 2.42E-22 |
| 105915239 | pim1 | Pim-1 proto-oncogene, serine/threonine kinase | 1136.432 | 0.491 | 3.09E-18 |
| 105940375 | LOC105940375 | tumor necrosis factor receptor superfamily member 6B-like | 77.147 | 0.366 | 1.86E-15 |
| 105915292 | LOC105915292 | uncharacterized LOC105915292 | 63.723 | 0.411 | 1.59E-14 |
| 105940207 | gadd45b | growth arrest and DNA damage inducible beta | 1978.343 | 0.450 | 2.15E-14 |
| 105917474 | LOC105917474 | protein L-Myc-1b-like | 413.508 | 0.397 | 2.15E-14 |
| 105938331 | LOC105938331 | transcription factor Sox-2-like | 64.025 | 0.448 | 2.64E-14 |
| 105937858 | LOC105937858 | protein FAM110A-like | 1288.418 | 0.327 | 1.01E-12 |
| 105922862 | ch25h | cholesterol 25-hydroxylase | 393.992 | 0.356 | 4.71E-12 |
| 105926298 | LOC105926298 | hairy/enhancer-of-split related with YRPW motif protein 1-like | 547.961 | 0.363 | 8.34E-12 |
| 105932163 | map3k8 | mitogen-activated protein kinase kinase kinase 8 | 367.315 | 0.416 | 1.38E-11 |
| 105925759 | fosl1 | FOS like 1, AP-1 transcription factor subunit | 183.442 | 0.342 | 3.03E-11 |
| 105937891 | irf1 | interferon regulatory factor 1 | 476.038 | 0.411 | 3.66E-11 |
| 105926891 | tnfrsf18 | TNF receptor superfamily member 18 | 359.978 | 0.410 | 7.54E-11 |
| 105919158 | slc39a1 | solute carrier family 39 member 1 | 214.719 | 0.403 | 1.65E-10 |
| 105926585 | dusp1 | dual specificity phosphatase 1 | 1099.648 | 0.376 | 1.92E-10 |
| 105932611 | fcrla | Fc receptor like A | 279.879 | 0.374 | 3.18E-10 |
| 105929553 | LOC105929553 | proto-oncogene c-Fos-like | 7008.199 | 0.348 | 4.46E-10 |
| 105940345 | LOC105940345 | SAM domain-containing protein SAMSN-1-like | 423.726 | 0.364 | 4.46E-10 |
| 105916408 | LOC105916408 | guanine nucleotide-binding protein subunit beta-5-like | 137.208 | 0.379 | 8.62E-10 |
| 105921752 | LOC105921752 | OX-2 membrane glycoprotein-like | 60.085 | 0.312 | 1.35E-09 |
| 105931158 | btg1 | BTG anti-proliferation factor 1 | 6685.677 | 0.290 | 1.49E-09 |
| 105935242 | nr4a1 | nuclear receptor subfamily 4 group A member 1 | 1300.217 | 0.251 | 2.30E-09 |
| 105919964 | LOC105919964 | early growth response protein 4-like | 2627.545 | 0.325 | 3.50E-09 |
| 105932456 | LOC105932456 | midnolin-like | 1757.166 | 0.288 | 4.19E-09 |
| 105919044 | egr1 | early growth response 1 | 6014.725 | 0.375 | 5.04E-09 |
| 105921046 | LOC105921046 | early growth response protein 1-like | 1951.515 | 0.340 | 7.16E-09 |
| 105931009 | klf2 | Kruppel like factor 2 | 1223.094 | 0.350 | 9.38E-09 |
| 105933664 | cish | cytokine inducible SH2 containing protein | 195.011 | 0.356 | 1.52E-08 |
| 105929554 | LOC105929554 | cyclin-dependent kinase inhibitor 1C-like | 220.362 | 0.366 | 1.52E-08 |
| 105919281 | LOC105919281 | myelin-oligodendrocyte glycoprotein-like | 55.612 | 0.327 | 1.55E-08 |
| 105916271 | gja4 | gap junction protein alpha 4 | 87.220 | 0.361 | 1.88E-08 |
| 105937076 | ier2 | immediate early response 2 | 1873.216 | 0.351 | 2.40E-08 |
| 105938432 | LOC105938432 | cysteine/serine-rich nuclear protein 1-like | 2357.002 | 0.356 | 4.49E-08 |
| 105938309 | LOC105938309 | transcription factor jun-D-like | 1742.187 | 0.333 | 4.92E-08 |
| 105915884 | LOC105915884 | C5a anaphylatoxin chemotactic receptor 1-like | 37.739 | 0.261 | 6.60E-08 |
| 105922834 | LOC105922834 | transcriptional regulator Myc-1-like | 355.703 | 0.296 | 6.60E-08 |
| 105915223 | ccng2 | cyclin G2 | 371.063 | 0.351 | 7.56E-08 |
| 105929572 | LOC105929572 | jun dimerization protein 2-like | 710.825 | 0.305 | 1.82E-07 |
| 105937041 | bhlhe40 | basic helix-loop-helix family member e40 | 5273.054 | 0.190 | 1.83E-07 |
| 105919975 | LOC105919975 | uncharacterized LOC105919975 | 300.431 | 0.312 | 2.45E-07 |
| 105920488 | LOC105920488 | tristetraprolin-like | 4237.383 | 0.306 | 4.34E-07 |
| 105930717 | rhob | ras homolog family member B | 201.996 | 0.331 | 4.57E-07 |
| 105918452 | ier5l | immediate early response 5 like | 614.355 | 0.279 | 1.08E-06 |
| 105917860 | LOC105917860 | uncharacterized LOC105917860 | 398.050 | 0.307 | 1.43E-06 |
| 105917553 | LOC105917553 | A-kinase anchor protein 12-like | 157.275 | 0.320 | 1.63E-06 |
| 105934661 | LOC105934661 | RING finger protein 186-like | 2552.834 | 0.287 | 1.84E-06 |
| 105919183 | ripk3 | receptor interacting serine/threonine kinase 3 | 130.757 | 0.309 | 2.63E-06 |
| 105918476 | irf2bp2 | interferon regulatory factor 2 binding protein 2 | 2949.972 | 0.191 | 2.77E-06 |
| 105926186 | zfp36l2 | ZFP36 ring finger protein like 2 | 339.408 | 0.276 | 3.16E-06 |
| 105916268 | LOC105916268 | gap junction beta-4 protein-like | 209.147 | 0.302 | 4.36E-06 |
| 105921524 | gpr34 | G protein-coupled receptor 34 | 250.751 | -0.302 | 7.25E-06 |
| 105930977 | dusp4 | dual specificity phosphatase 4 | 804.246 | 0.303 | 1.04E-05 |
| 105920383 | LOC105920383 | interleukin-12 receptor subunit beta-2-like | 149.787 | 0.297 | 1.77E-05 |
| 105916346 | rrad | RRAD, Ras related glycolysis inhibitor and calcium channel regulator | 995.510 | 0.271 | 2.04E-05 |
| 105916409 | bcl2l10 | BCL2 like 10 | 258.419 | 0.278 | 3.62E-05 |
| 105940343 | LOC105940343 | uncharacterized LOC105940343 | 95.699 | 0.283 | 3.62E-05 |
| 105933498 | socs1 | suppressor of cytokine signaling 1 | 111.865 | 0.282 | 5.16E-05 |
| 105934239 | LOC105934239 | class E basic helix-loop-helix protein 40-like | 1348.081 | 0.157 | 6.09E-05 |
| 105935283 | LOC105935283 | CCAAT/enhancer-binding protein beta-like | 1632.978 | 0.274 | 6.74E-05 |
| 105918977 | LOC105918977 | transcriptional regulator Myc-2-like | 282.571 | 0.281 | 7.48E-05 |
| 105924198 | LOC105924198 | uncharacterized LOC105924198 | 183.155 | 0.275 | 8.23E-05 |
| 105915941 | LOC105915941 | growth arrest and DNA damage-inducible protein GADD45 alpha-like | 234.009 | 0.273 | 9.26E-05 |
| 105930719 | tagap | T-cell activation RhoGTPase activating protein | 1129.167 | 0.204 | 0.000117 |
| 105920633 | ankrd1 | ankyrin repeat domain 1 | 139.518 | 0.273 | 0.000119 |
| 105916702 | dennd4a | DENN domain containing 4A | 1941.515 | 0.161 | 0.000166 |
| 105935734 | LOC105935734 | rho GTPase-activating protein 21-like | 1194.901 | 0.162 | 0.000185 |
| 105915527 | LOC105915527 | claudin-5-like | 153.408 | 0.269 | 0.000197 |
| 105926672 | LOC105926672 | ADM2-like | 39.637 | 0.226 | 0.000234 |
| 105924534 | LOC105924534 | C-X-C motif chemokine 10-like | 179.374 | 0.267 | 0.000250 |
| 105926318 | nfkbid | NFKB inhibitor delta | 20.786 | 0.166 | 0.000253 |
| 105918808 | ddit4 | DNA damage inducible transcript 4 | 3281.743 | -0.238 | 0.000291 |
| 105925695 | npas4 | neuronal PAS domain protein 4 | 3069.379 | 0.222 | 0.000291 |
| 105934939 | LOC105934939 | uncharacterized LOC105934939 | 59.381 | 0.240 | 0.000379 |
| 105920039 | LOC105920039 | protein BTG3-like | 1407.280 | 0.214 | 0.000384 |
| 105937034 | gpr84 | G protein-coupled receptor 84 | 90.847 | 0.252 | 0.000392 |
| 105929477 | LOC105929477 | proto-oncogene c-Fos-like | 757.593 | 0.224 | 0.000452 |
| 105932356 | LOC105932356 | mucosa-associated lymphoid tissue lymphoma translocation protein 1-like | 313.039 | 0.239 | 0.000640 |
| 105921394 | LOC105921394 | N-chimaerin-like | 1372.628 | 0.197 | 0.000666 |
| 105934850 | rel | REL proto-oncogene, NF-kB subunit | 175.232 | 0.250 | 0.000724 |
| 105940351 | LOC105940351 | protein phosphatase Slingshot homolog 1-like | 386.602 | 0.235 | 0.000971 |
| 105928786 | LOC105928786 | delphilin-like | 2443.494 | 0.217 | 0.001168 |
| 105929362 | LOC105929362 | uncharacterized LOC105929362 | 12.812 | 0.136 | 0.001168 |
| 105918420 | per1 | period circadian clock 1 | 1823.664 | 0.151 | 0.001217 |
| 105921053 | LOC105921053 | calmin-like | 2496.644 | 0.172 | 0.001266 |
| 105925309 | LOC105925309 | transcription factor AP-1-like | 752.590 | 0.232 | 0.001552 |
| 105931951 | LOC105931951 | tubulin alpha-3 chain-like | 5818.511 | -0.087 | 0.001737 |
| 105921799 | LOC105921799 | cell wall integrity and stress response component 1-like | 21.195 | 0.141 | 0.001795 |
| 105916030 | basp1 | brain abundant membrane attached signal protein 1 | 3827.133 | 0.107 | 0.002037 |
| 105935310 | grasp | general receptor for phosphoinositides 1 associated scaffold protein | 311.816 | 0.239 | 0.002192 |
| 105917541 | otx2 | orthodenticle homeobox 2 | 1211.389 | 0.209 | 0.002258 |
| 105917570 | LOC105917570 | fructose-bisphosphate aldolase A | 32.172 | 0.115 | 0.002692 |
| 105938021 | LOC105938021 | striated muscle preferentially expressed protein kinase-like | 1176.461 | 0.232 | 0.002763 |
| 105932983 | LOC105932983 | parvalbumin, thymic CPV3-like | 14.444 | -0.082 | 0.003307 |
| 105930300 | LOC105930300 | SH3 and multiple ankyrin repeat domains protein 3-like | 885.756 | 0.195 | 0.003331 |
| 105919110 | LOC105919110 | heat shock 70 kDa protein 1 | 117.473 | 0.224 | 0.004575 |
| 105929424 | LOC105929424 | connector enhancer of kinase suppressor of ras 2-like | 1371.547 | 0.169 | 0.004579 |
| 105917657 | LOC105917657 | G-protein coupled receptor 56-like | 185.495 | 0.224 | 0.005810 |
| 105937090 | heg1 | heart development protein with EGF like domains 1 | 1256.595 | 0.168 | 0.007297 |
| 105930425 | LOC105930425 | tetraspanin-33-like | 3047.843 | 0.161 | 0.007297 |
| 105921202 | LOC105921202 | protein c-ets-2-A-like | 189.578 | 0.211 | 0.007669 |
| 105921723 | lpar3 | lysophosphatidic acid receptor 3 | 19.251 | 0.120 | 0.008917 |
| 105930718 | LOC105930718 | uncharacterized LOC105930718 | 233.544 | 0.212 | 0.012242 |
| 105934829 | LOC105934829 | testisin-like | 13.580 | -0.072 | 0.012414 |
| 105915875 | asmt | acetylserotonin O-methyltransferase | 29.450 | -0.115 | 0.015782 |
| 105925184 | LOC105925184 | transferrin receptor protein 1-like | 384.859 | 0.193 | 0.016050 |
| 105938544 | reln | reelin | 3163.676 | 0.156 | 0.016526 |
| 105926275 | LOC105926275 | protein phosphatase 1 regulatory subunit 15B-like | 1485.248 | 0.152 | 0.017634 |
| 105925384 | cdkl5 | cyclin dependent kinase like 5 | 2944.273 | 0.121 | 0.020191 |
| 105938829 | tp53bp1 | tumor protein p53 binding protein 1 | 1509.610 | 0.135 | 0.020581 |
| 105926696 | LOC105926696 | BTB/POZ domain-containing protein KCTD5-like | 1021.009 | 0.143 | 0.021574 |
| 105915482 | LOC105915482 | protein bassoon-like | 4249.688 | 0.126 | 0.023477 |
| 105936954 | mpl | MPL proto-oncogene, thrombopoietin receptor | 63.301 | 0.189 | 0.023477 |
| 105925977 | kbtbd11 | kelch repeat and BTB domain containing 11 | 2067.854 | 0.160 | 0.024859 |
| 105926698 | LOC105926698 | carbonic anhydrase 4-like | 15.974 | -0.088 | 0.024938 |
| 105926120 | LOC105926120 | dedicator of cytokinesis protein 3-like | 6850.888 | 0.160 | 0.026226 |
| 105918379 | LOC105918379 | receptor-type tyrosine-protein phosphatase U-like | 1737.762 | 0.151 | 0.026927 |
| 105937019 | LOC105937019 | disco-interacting protein 2 homolog B-A-like | 2567.617 | 0.090 | 0.026927 |
| 105934707 | tiparp | TCDD inducible poly(ADP-ribose) polymerase | 1040.660 | 0.143 | 0.027950 |
| 105919230 | LOC105919230 | type III iodothyronine deiodinase-like | 76.804 | 0.196 | 0.030813 |
| 105933249 | nfia | nuclear factor I A | 437.599 | 0.183 | 0.031456 |
| 105920771 | LOC105920771 | early growth response protein 2b-like | 278.921 | 0.114 | 0.032737 |
| 105938769 | LOC105938769 | disks large-associated protein 2-like | 676.264 | 0.177 | 0.033468 |
| 105926147 | kndc1 | kinase non-catalytic C-lobe domain containing 1 | 891.431 | 0.186 | 0.034518 |
| 105915796 | LOC105915796 | plasma membrane calcium-transporting ATPase 1-like | 5928.587 | 0.133 | 0.035605 |
| 105916754 | LOC105916754 | glutathione S-transferase A-like | 8579.019 | -0.088 | 0.036830 |
| 105933437 | LOC105933437 | sal-like protein 3 | 1934.592 | -0.101 | 0.036830 |
| 105920010 | brd2 | bromodomain containing 2 | 3467.765 | 0.121 | 0.039172 |
| 105915730 | LOC105915730 | parvalbumin, thymic CPV3-like | 7.685 | -0.061 | 0.040555 |
| 105933548 | LOC105933548 | interleukin-3 receptor class 2 subunit beta-like | 258.282 | 0.189 | 0.040975 |
| 105923962 | LOC105923962 | uncharacterized LOC105923962 | 1354.547 | 0.143 | 0.043156 |
| 105933159 | camsap2 | calmodulin regulated spectrin associated protein family member 2 | 2143.868 | 0.105 | 0.044432 |
| 105933463 | eif4g2 | eukaryotic translation initiation factor 4 gamma 2 | 3314.614 | 0.153 | 0.044571 |
| 105925488 | LOC105925488 | uncharacterized LOC105925488 | 31931.708 | -0.086 | 0.047764 |
| 105940360 | LOC105940360 | lysine-specific demethylase 6B-like | 2508.826 | 0.142 | 0.048877 |
| 105918714 | LOC105918714 | IQ motif and SEC7 domain-containing protein 2-like | 1741.103 | 0.155 | 0.051125 |
| 105934948 | clec2d | C-type lectin domain family 2 member D | 4512.247 | -0.098 | 0.055670 |
| 105915216 | gcnt7 | glucosaminyl (N-acetyl) transferase family member 7 | 295.253 | 0.187 | 0.055868 |
| 105932322 | LOC105932322 | C2 calcium-dependent domain-containing protein 4C-like | 168.825 | -0.171 | 0.055868 |
| 105934888 | LOC105934888 | cell surface glycoprotein CD200 receptor 1-B-like | 35.796 | 0.153 | 0.057238 |
| 105919795 | LOC105919795 | serine/threonine-protein phosphatase 2B catalytic subunit alpha isoform-like | 2525.532 | 0.157 | 0.058486 |
| 105937083 | LOC105937083 | microtubule-associated protein 2-like | 10602.780 | 0.142 | 0.058486 |
| 105927279 | rho | rhodopsin | 48.187 | -0.107 | 0.058486 |
| 105935641 | LOC105935641 | double-stranded RNA-specific editase 1-like | 1956.495 | 0.117 | 0.062899 |
| 105915302 | LOC105915302 | P-selectin glycoprotein ligand 1-like | 83.605 | 0.182 | 0.065916 |
| 105920114 | bag3 | BCL2 associated athanogene 3 | 380.913 | 0.155 | 0.065932 |
| 105926556 | nlrc5 | NLR family CARD domain containing 5 | 371.723 | 0.183 | 0.065932 |
| 105934912 | kmt2c | lysine methyltransferase 2C | 2925.203 | 0.120 | 0.072075 |
| 105936919 | syde2 | synapse defective Rho GTPase homolog 2 | 1118.549 | 0.138 | 0.072106 |
| 105929455 | ylpm1 | YLP motif containing 1 | 3002.433 | 0.088 | 0.073472 |
| 105935516 | LOC105935516 | BTB/POZ domain-containing protein 6-B-like | 1716.530 | 0.127 | 0.073750 |
| 105921490 | zc3h13 | zinc finger CCCH-type containing 13 | 2228.354 | 0.107 | 0.073753 |
| 105937267 | add2 | adducin 2 | 3711.026 | 0.078 | 0.077896 |
| 105940279 | lcp1 | lymphocyte cytosolic protein 1 | 1550.305 | -0.137 | 0.080872 |
| 105916679 | LOC105916679 | period circadian protein homolog 2-like | 664.433 | 0.162 | 0.082367 |
| 105922911 | tjap1 | tight junction associated protein 1 | 216.050 | 0.171 | 0.086417 |
| 105916803 | hamp | hepcidin antimicrobial peptide | 42.525 | 0.120 | 0.087861 |
| 105933619 | rin1 | Ras and Rab interactor 1 | 816.992 | 0.173 | 0.087861 |
| 105929373 | LOC105929373 | glutamate receptor 3-like | 5838.048 | 0.146 | 0.087991 |
| 105915764 | LOC105915764 | lymphotoxin-alpha-like | 307.574 | 0.168 | 0.088290 |
| 105926141 | LOC105926141 | B-cell linker protein-like | 128.249 | 0.178 | 0.088545 |
| 105933523 | LOC105933523 | lipopolysaccharide-induced tumor necrosis factor-alpha factor homolog | 5.965 | -0.051 | 0.088912 |
| 105916427 | tmem198 | transmembrane protein 198 | 955.498 | 0.125 | 0.088912 |
| 105918207 | phlda2 | pleckstrin homology like domain family A member 2 | 266.546 | 0.160 | 0.088982 |
| 105926253 | LOC105926253 | switch-associated protein 70-like | 2286.079 | -0.102 | 0.090418 |
| 105917949 | LOC105917949 | double C2-like domain-containing protein beta | 320.029 | 0.173 | 0.090609 |
| 105937030 | LOC105937030 | inositol 1,4,5-trisphosphate receptor type 1-like | 726.529 | 0.145 | 0.090609 |
| 105921661 | rapgefl1 | Rap guanine nucleotide exchange factor like 1 | 1887.116 | 0.145 | 0.090609 |
| 105917557 | LOC105917557 | double C2-like domain-containing protein alpha | 997.077 | 0.165 | 0.090641 |
| 105929497 | LOC105929497 | potassium voltage-gated channel subfamily H member 5-like | 107.213 | 0.172 | 0.090641 |
| 105926634 | LOC105926634 | rootletin-like | 6797.168 | 0.171 | 0.093954 |
| 105933165 | LOC105933165 | GRAM domain-containing protein 3-like | 1274.354 | 0.161 | 0.095407 |
| 105917779 | mef2d | myocyte enhancer factor 2D | 1553.046 | 0.146 | 0.095407 |
| 105939497 | LOC105939497 | bromodomain-containing protein 4-like | 1559.429 | 0.100 | 0.097798 |
| 105935586 | bhlhe41 | basic helix-loop-helix family member e41 | 1152.492 | 0.143 | 0.099438 |
| 105917939 | usmg5 | up-regulated during skeletal muscle growth 5 homolog (mouse) | 1783.672 | -0.096 | 0.099441 |
| 105927142 | nuak2 | NUAK family kinase 2 | 344.624 | 0.166 | 0.100755 |
| 105915455 | LOC105915455 | chromosome unknown open reading frame, human C8orf4 | 968.939 | 0.137 | 0.105478 |
| 105917992 | ep300 | E1A binding protein p300 | 1387.632 | 0.098 | 0.106574 |
| 105932851 | foxf2 | forkhead box F2 | 228.311 | 0.165 | 0.106574 |
| 105920003 | trpv1 | transient receptor potential cation channel subfamily V member 1 | 80.526 | -0.173 | 0.107073 |
| 105938788 | herc1 | HECT and RLD domain containing E3 ubiquitin protein ligase family member 1 | 5462.945 | 0.118 | 0.108600 |
| 105925999 | LOC105925999 | probable G-protein coupled receptor 22 | 299.589 | 0.163 | 0.111529 |
| 105916583 | LOC105916583 | inverted formin-2-like | 2004.048 | 0.145 | 0.112604 |
| 105933344 | LOC105933344 | N-acetyllactosaminide beta-1,3-N-acetylglucosaminyltransferase 2-like | 223.062 | 0.168 | 0.112604 |
| 105921165 | LOC105921165 | probable E3 ubiquitin-protein ligase HERC1 | 5037.614 | 0.100 | 0.113078 |
| 105930872 | LOC105930872 | transcription factor Sox-9-A-like | 280.230 | -0.172 | 0.113078 |
| 105932712 | LOC105932712 | uncharacterized LOC105932712 | 759.158 | 0.122 | 0.115342 |
| 105938284 | LOC105938284 | atypical chemokine receptor 3-like | 1534.331 | -0.107 | 0.120516 |
| 105920253 | LOC105920253 | macrophage mannose receptor 1-like | 638.117 | -0.131 | 0.122544 |
| 105920693 | hnrnpa0 | heterogeneous nuclear ribonucleoprotein A0 | 9537.359 | 0.088 | 0.125822 |
| 105917734 | LOC105917734 | carbohydrate sulfotransferase 1-like | 283.844 | 0.168 | 0.127257 |
| 105915909 | mafk | MAF bZIP transcription factor K | 100.528 | 0.161 | 0.132836 |
| 105916117 | LOC105916117 | transcription factor HES-4-B-like | 210.046 | 0.168 | 0.134689 |
| 105921084 | vps13c | vacuolar protein sorting 13 homolog C | 3329.058 | 0.094 | 0.135269 |
| 105917844 | birc6 | baculoviral IAP repeat containing 6 | 2856.172 | 0.094 | 0.138567 |
| 105934871 | pnrc2 | proline rich nuclear receptor coactivator 2 | 7255.573 | 0.142 | 0.140903 |
| 105917607 | fam46c | family with sequence similarity 46 member C | 46.670 | 0.141 | 0.142524 |
| 105935026 | cep126 | centrosomal protein 126 | 261.683 | 0.167 | 0.145667 |
| 105918326 | LOC105918326 | mucin-12-like | 1852.146 | 0.134 | 0.147689 |
| 105928677 | LOC105928677 | cadherin-23-like | 236.806 | 0.159 | 0.149672 |
| 105921156 | gnmt | glycine N-methyltransferase | 114.400 | 0.166 | 0.152111 |
| 105918418 | LOC105918418 | ammonium transporter Rh type C 2-like | 23.825 | -0.079 | 0.152111 |
| 105936782 | LOC105936782 | methylcytosine dioxygenase TET3-like | 1563.272 | 0.116 | 0.152138 |
| 105921658 | LOC105921658 | nucleoside diphosphate kinase B-like | 29.486 | 0.053 | 0.156921 |
| 105916319 | cacna1c | calcium voltage-gated channel subunit alpha1 C | 2195.711 | 0.123 | 0.159607 |
| 105932804 | LOC105932804 | hepatic leukemia factor-like | 1466.864 | 0.115 | 0.159607 |
| 105920092 | vps13d | vacuolar protein sorting 13 homolog D | 1554.298 | 0.105 | 0.159607 |
| 105922878 | LOC105922878 | galactose-specific lectin nattectin-like | 22.228 | -0.061 | 0.160102 |
| 105933840 | entpd1 | ectonucleoside triphosphate diphosphohydrolase 1 | 666.602 | 0.128 | 0.167099 |
| 105934556 | LOC105934556 | protein unc-13 homolog A-like | 916.084 | 0.124 | 0.168406 |
| 105932528 | r3hdm1 | R3H domain containing 1 | 11361.425 | 0.120 | 0.168406 |
| 105932396 | syne1 | spectrin repeat containing nuclear envelope protein 1 | 11568.959 | 0.120 | 0.169779 |
| 105925743 | LOC105925743 | PCTP-like protein | 21.743 | -0.065 | 0.171530 |
| 105932116 | camkk2 | calcium/calmodulin dependent protein kinase kinase 2 | 2147.657 | 0.125 | 0.171749 |
| 105933111 | LOC105933111 | claudin-4-like | 7.593 | -0.053 | 0.174221 |
| 105933056 | LOC105933056 | uncharacterized LOC105933056 | 609.787 | 0.142 | 0.176453 |
| 105927082 | LOC105927082 | protein FAM212A-like | 109.780 | -0.162 | 0.179817 |
| 105929372 | LOC105929372 | interleukin-1 receptor accessory protein-like | 203.212 | 0.158 | 0.186656 |
| 105938806 | LOC105938806 | tetraspanin-18-like | 382.703 | 0.158 | 0.186925 |
| 105920979 | dock3 | dedicator of cytokinesis 3 | 1974.432 | 0.112 | 0.187609 |
| 105935206 | iqsec2 | IQ motif and Sec7 domain 2 | 5609.368 | 0.161 | 0.187609 |
| 105928753 | map1b | microtubule associated protein 1B | 6530.475 | -0.078 | 0.187609 |
| 105919299 | LOC105919299 | sprouty-related, EVH1 domain-containing protein 2-like | 395.055 | 0.135 | 0.191235 |
| 105924533 | LOC105924533 | uncharacterized LOC105924533 | 20.871 | -0.111 | 0.191235 |
| 105936936 | raver2 | ribonucleoprotein, PTB binding 2 | 878.051 | 0.151 | 0.191235 |
| 105934832 | LOC105934832 | serine protease 27-like | 8.190 | -0.047 | 0.195133 |
| 105928462 | n4bp3 | NEDD4 binding protein 3 | 809.872 | 0.151 | 0.195556 |
| 105915550 | LOC105915550 | G-protein coupled receptor 12-like | 382.083 | 0.136 | 0.197192 |
| 105918318 | psme1 | proteasome activator subunit 1 | 640.527 | -0.124 | 0.202621 |
| 105915271 | csf1r | colony stimulating factor 1 receptor | 185.944 | -0.156 | 0.203279 |
| 105925586 | LOC105925586 | rho-associated protein kinase 2-like | 1311.995 | 0.145 | 0.203279 |
| 105926067 | LOC105926067 | glutamate receptor-interacting protein 2-like | 438.976 | 0.147 | 0.204655 |
| 105919051 | ckm | creatine kinase, M-type | 31.084 | 0.045 | 0.206621 |
| 105930949 | pgam2 | phosphoglycerate mutase 2 | 16.437 | 0.079 | 0.208320 |
| 105938175 | dapk1 | death associated protein kinase 1 | 3040.763 | 0.127 | 0.211659 |
| 105926735 | fam131a | family with sequence similarity 131 member A | 1299.388 | 0.136 | 0.211659 |
| 105916598 | dmxl2 | Dmx like 2 | 9412.084 | 0.088 | 0.212590 |
| 105938277 | LOC105938277 | uncharacterized LOC105938277 | 362.362 | 0.136 | 0.213019 |
| 105924082 | LOC105924082 | uncharacterized LOC105924082 | 153.872 | -0.157 | 0.213664 |
| 105935296 | LOC105935296 | pancreatic progenitor cell differentiation and proliferation factor A-like | 65.275 | -0.127 | 0.213664 |
| 105919965 | LOC105919965 | steroid 17-alpha-hydroxylase/17,20 lyase-like | 223.172 | 0.155 | 0.214644 |
| 105936962 | LOC105936962 | ephrin type-B receptor 1-like | 598.238 | 0.130 | 0.214644 |
| 105933410 | mbp | myelin basic protein | 3126.294 | -0.145 | 0.214988 |
| 105938584 | LOC105938584 | pyruvate kinase PKM-like | 9.444 | 0.059 | 0.216216 |
| 105935735 | arhgap21 | Rho GTPase activating protein 21 | 2797.200 | 0.093 | 0.217006 |
| 105916328 | slc22a31 | solute carrier family 22 member 31 | 65.019 | 0.152 | 0.218719 |
| 105916608 | mapk6 | mitogen-activated protein kinase 6 | 3688.411 | 0.076 | 0.218898 |
| 105925713 | LOC105925713 | phosphatidylinositol 3,4,5-trisphosphate 5-phosphatase 2B-like | 1418.554 | 0.144 | 0.221170 |
| 105926751 | serpine2 | serpin family E member 2 | 3298.905 | -0.092 | 0.221170 |
| 105915513 | ksr2 | kinase suppressor of ras 2 | 676.855 | 0.146 | 0.221635 |
| 105918343 | golgb1 | golgin B1 | 1613.276 | 0.088 | 0.223623 |
| 105930905 | dusp7 | dual specificity phosphatase 7 | 854.027 | 0.112 | 0.223985 |
| 105915567 | LOC105915567 | phosphatidylinositol 3-kinase regulatory subunit alpha | 824.226 | -0.114 | 0.223985 |
| 105933351 | LOC105933351 | 1-phosphatidylinositol 4,5-bisphosphate phosphodiesterase beta-1-like | 7503.247 | 0.154 | 0.224519 |
| 105932812 | mylpf | myosin light chain, phosphorylatable, fast skeletal muscle | 125.622 | 0.126 | 0.224604 |
| 105932156 | kcnh2 | potassium voltage-gated channel subfamily H member 2 | 796.712 | 0.138 | 0.225425 |
| 105938427 | gyg1 | glycogenin 1 | 11236.385 | -0.119 | 0.225492 |
| 105915670 | LOC105915670 | leukocyte surface antigen CD53-like | 516.265 | 0.138 | 0.225492 |
| 105932314 | LOC105932314 | ras-specific guanine nucleotide-releasing factor 1 | 2803.723 | 0.095 | 0.225492 |
| 105937902 | tectb | tectorin beta | 48.185 | -0.099 | 0.225934 |
| 105925766 | LOC105925766 | rod cGMP-specific 3',5'-cyclic phosphodiesterase subunit alpha-like | 15.203 | -0.076 | 0.231800 |
| 105920811 | LOC105920811 | AP-1 complex subunit sigma-2-like | 2940.263 | -0.082 | 0.231829 |
| 105930728 | LOC105930728 | potassium voltage-gated channel subfamily F member 1-like | 662.787 | 0.140 | 0.232274 |
| 105917938 | LOC105917938 | alpha-internexin-like | 3564.079 | -0.091 | 0.232490 |
| 105919129 | psmc4 | proteasome 26S subunit, ATPase 4 | 1754.099 | -0.079 | 0.232490 |
| 105921513 | adamts1 | ADAM metallopeptidase with thrombospondin type 1 motif 1 | 192.335 | 0.154 | 0.232789 |
| 105940209 | LOC105940209 | cocaine- and amphetamine-regulated transcript protein-like | 116.333 | -0.135 | 0.232944 |
| 105918233 | cd44 | CD44 molecule (Indian blood group) | 2012.479 | -0.108 | 0.236650 |
| 105925861 | LOC105925861 | tyrosine-protein phosphatase non-receptor type 11-like | 396.331 | 0.150 | 0.239364 |
| 105935821 | LOC105935821 | sodium-dependent phosphate transporter 1-B-like | 1856.823 | 0.114 | 0.239438 |
| 105938191 | LOC105938191 | serine/threonine-protein kinase WNK1-like | 3498.507 | 0.079 | 0.239438 |
| 105931131 | LOC105931131 | proteinase-activated receptor 1-like | 2721.007 | 0.149 | 0.240241 |
| 105936970 | cyr61 | cysteine rich angiogenic inducer 61 | 812.931 | 0.142 | 0.245677 |
| 105916592 | LOC105916592 | pleckstrin homology domain-containing family G member 3-like | 1569.436 | 0.151 | 0.245678 |
| 105930854 | LOC105930854 | histone acetyltransferase p300-like | 1683.992 | 0.090 | 0.249343 |
| 105926373 | LOC105926373 | uncharacterized LOC105926373 | 310.753 | -0.131 | 0.249562 |
| 105932506 | tanc2 | tetratricopeptide repeat, ankyrin repeat and coiled-coil containing 2 | 3912.836 | 0.114 | 0.249847 |
| 105926071 | zbtb46 | zinc finger and BTB domain containing 46 | 483.945 | 0.151 | 0.249847 |
| 105918159 | cacnb2 | calcium voltage-gated channel auxiliary subunit beta 2 | 2594.281 | 0.131 | 0.252076 |
| 105933191 | misp | mitotic spindle positioning | 105.744 | 0.128 | 0.252987 |
| 105921488 | LOC105921488 | probable ribonuclease ZC3H12C | 848.755 | 0.125 | 0.253385 |
| 105933666 | LOC105933666 | ephrin type-A receptor 2-like | 250.643 | 0.151 | 0.253385 |
| 105934533 | LOC105934533 | band 4.1-like protein 1 | 2999.646 | 0.096 | 0.256549 |
| 105917773 | LOC105917773 | uncharacterized LOC105917773 | 11.187 | 0.056 | 0.260106 |
| 105915259 | nr4a3 | nuclear receptor subfamily 4 group A member 3 | 894.569 | 0.105 | 0.260156 |
| 105934907 | LOC105934907 | serine/threonine-protein kinase SIK2-like | 1032.501 | 0.148 | 0.262008 |
| 105921519 | ccng1 | cyclin G1 | 12574.709 | -0.074 | 0.263809 |
| 105932547 | LOC105932547 | protein sprouty homolog 2-like | 1155.782 | -0.103 | 0.265277 |
| 105918492 | camk2a | calcium/calmodulin dependent protein kinase II alpha | 9925.696 | 0.148 | 0.271932 |
| 105919212 | LOC105919212 | uncharacterized LOC105919212 | 5.090 | -0.055 | 0.271932 |
| 105937864 | LOC105937864 | cytochrome c oxidase subunit 4 isoform 2, mitochondrial-like | 3171.671 | -0.085 | 0.271932 |
| 105920992 | gp1bb | glycoprotein Ib platelet beta subunit | 36.272 | 0.100 | 0.273415 |
| 105937089 | LOC105937089 | myosin light chain 1, skeletal muscle isoform | 12.249 | 0.064 | 0.273415 |
| 105916195 | atp6v1f | ATPase H+ transporting V1 subunit F | 3181.467 | -0.080 | 0.280844 |
| 105915377 | cabin1 | calcineurin binding protein 1 | 1423.848 | 0.097 | 0.283591 |
| 105930970 | slc46a2 | solute carrier family 46 member 2 | 329.841 | 0.146 | 0.283591 |
| 105935146 | LOC105935146 | brain-specific angiogenesis inhibitor 1-like | 9511.222 | 0.139 | 0.284382 |
| 105931007 | ptprf | protein tyrosine phosphatase, receptor type F | 2875.913 | 0.086 | 0.285332 |
| 105925219 | LOC105925219 | leucine rich adaptor protein 1-like | 76.176 | 0.134 | 0.285975 |
| 105932894 | trim9 | tripartite motif containing 9 | 1035.348 | 0.121 | 0.286433 |
| 105931087 | LOC105931087 | cholesterol 25-hydroxylase-like protein 2 | 15.666 | -0.072 | 0.288317 |
| 105932418 | LOC105932418 | tubby-related protein 4-like | 6004.186 | 0.116 | 0.288317 |
| 105930979 | LOC105930979 | sorting nexin-18-like | 239.003 | 0.147 | 0.288592 |
| 105932917 | pygl | glycogen phosphorylase L | 2440.513 | -0.068 | 0.288592 |
| 105935004 | fam181b | family with sequence similarity 181 member B | 1006.094 | 0.117 | 0.288677 |
| 105921218 | crtam | cytotoxic and regulatory T-cell molecule | 127.671 | 0.146 | 0.290312 |
| 105920509 | LOC105920509 | ras and Rab interactor 3-like | 102.505 | 0.146 | 0.296489 |
| 105923769 | LOC105923769 | connector enhancer of kinase suppressor of ras 2-like | 418.735 | 0.135 | 0.298257 |
| 105926591 | wee2 | WEE1 homolog 2 | 56.152 | 0.122 | 0.301323 |
| 105926766 | LOC105926766 | large neutral amino acids transporter small subunit 4-like | 1517.165 | 0.093 | 0.301991 |
| 105916586 | LOC105916586 | inverted formin-2-like | 587.612 | 0.136 | 0.302374 |
| 105940390 | mtmr3 | myotubularin related protein 3 | 1494.191 | 0.096 | 0.303334 |
| 105915582 | LOC105915582 | disks large-associated protein 1-like | 588.202 | 0.131 | 0.304299 |
| 105919114 | LOC105919114 | forkhead box protein J1-A-like | 292.609 | 0.142 | 0.304941 |
| 105918046 | LOC105918046 | poly(rC)-binding protein 3-like | 49.448 | -0.135 | 0.310139 |
| 105915548 | LOC105915548 | wiskott-Aldrich syndrome protein family member 3-like | 2373.930 | 0.090 | 0.310660 |
| 105940200 | camk4 | calcium/calmodulin dependent protein kinase IV | 1555.032 | 0.101 | 0.315054 |
| 105934985 | shisa7 | shisa family member 7 | 5726.878 | 0.114 | 0.318163 |
| 105936257 | tbkbp1 | TBK1 binding protein 1 | 671.009 | 0.124 | 0.321732 |
| 105940281 | itm2b | integral membrane protein 2B | 6496.499 | -0.081 | 0.322021 |
| 105925206 | LOC105925206 | regulator of G-protein signaling 5-like | 149.928 | 0.121 | 0.322187 |
| 105934233 | LOC105934233 | copine-9-like | 3197.077 | 0.123 | 0.322187 |
| 105917704 | LOC105917704 | cocaine- and amphetamine-regulated transcript protein-like | 126.709 | -0.126 | 0.323985 |
| 105925463 | LOC105925463 | homer protein homolog 1-like | 1005.076 | 0.120 | 0.323985 |
| 105938069 | grin2b | glutamate ionotropic receptor NMDA type subunit 2B | 1999.886 | 0.115 | 0.328355 |
| 105930789 | kiaa2026 | KIAA2026 ortholog | 800.074 | 0.102 | 0.328440 |
| 105938299 | arrdc2 | arrestin domain containing 2 | 899.177 | -0.115 | 0.333306 |
| 105928050 | cfap44 | cilia and flagella associated protein 44 | 769.979 | -0.107 | 0.333306 |
| 105915427 | LOC105915427 | troponin T, fast skeletal muscle isoforms-like | 27.610 | 0.091 | 0.333867 |
| 105935149 | LOC105935149 | ras/Rap GTPase-activating protein SynGAP-like | 1601.277 | 0.120 | 0.333867 |
| 105918488 | LOC105918488 | A disintegrin and metalloproteinase with thrombospondin motifs 2-like | 17.461 | -0.089 | 0.335631 |
| 105940358 | LOC105940358 | uncharacterized LOC105940358 | 15.014 | -0.046 | 0.335631 |
| 105920174 | sptbn1 | spectrin beta, non-erythrocytic 1 | 14141.422 | 0.074 | 0.335631 |
| 105933687 | cacna2d2 | calcium voltage-gated channel auxiliary subunit alpha2delta 2 | 1948.165 | 0.123 | 0.336444 |
| 105926328 | kmt2b | lysine methyltransferase 2B | 954.213 | 0.097 | 0.336444 |
| 105934655 | LOC105934655 | regulator of G-protein signaling 12-like | 695.718 | 0.125 | 0.336617 |
| 105916806 | vsig10l | V-set and immunoglobulin domain containing 10 like | 5.102 | -0.040 | 0.336642 |
| 105938271 | dopey2 | dopey family member 2 | 3537.341 | 0.067 | 0.337446 |
| 105918009 | plbd1 | phospholipase B domain containing 1 | 188.282 | -0.138 | 0.337665 |
| 105915304 | LOC105915304 | iron-sulfur cluster assembly enzyme ISCU, mitochondrial-like | 8090.154 | -0.060 | 0.338913 |
| 105938253 | c1qa | complement C1q A chain | 310.528 | -0.129 | 0.339462 |
| 105926771 | epha4 | EPH receptor A4 | 4131.754 | 0.096 | 0.340009 |
| 105919001 | LOC105919001 | ADP-ribosyl cyclase/cyclic ADP-ribose hydrolase 1-like | 227.609 | -0.137 | 0.340009 |
| 105932938 | LOC105932938 | desumoylating isopeptidase 2-like | 503.568 | 0.124 | 0.340009 |
| 105935124 | LOC105935124 | protein S100-A1-like | 44.045 | -0.074 | 0.340009 |
| 105937854 | LOC105937854 | collagen alpha-1(XIV) chain-like | 336.840 | 0.127 | 0.340009 |
| 105918070 | lrch1 | leucine rich repeats and calponin homology domain containing 1 | 574.595 | 0.121 | 0.340009 |
| 105925864 | mmp23b | matrix metallopeptidase 23B | 81.107 | 0.120 | 0.341080 |
| 105933388 | pou3f2 | POU class 3 homeobox 2 | 379.178 | -0.117 | 0.341352 |
| 105935279 | slc48a1 | solute carrier family 48 member 1 | 1159.227 | -0.087 | 0.341458 |
| 105916647 | LOC105916647 | regulator of G-protein signaling 21-like | 654.803 | 0.140 | 0.346744 |
| 105920923 | LOC105920923 | F-box/LRR-repeat protein 19-like | 886.472 | 0.102 | 0.348000 |
| 105933385 | LOC105933385 | leucine-rich repeat-containing protein 32-like | 8.908 | 0.067 | 0.348000 |
| 105925570 | rasgrp2 | RAS guanyl releasing protein 2 | 1054.194 | 0.110 | 0.348000 |
| 105930894 | LOC105930894 | phosphatidylinositol 5-phosphate 4-kinase type-2 gamma-like | 1181.054 | -0.085 | 0.349415 |
| 105925345 | atp1b1 | ATPase Na+/K+ transporting subunit beta 1 | 7067.064 | -0.058 | 0.349984 |
| 105916286 | dagla | diacylglycerol lipase alpha | 4784.586 | 0.101 | 0.349984 |
| 105932283 | LOC105932283 | cadherin-like protein 26 | 3.285 | -0.033 | 0.349984 |
| 105935794 | LOC105935794 | FUN14 domain-containing protein 1-like | 2684.682 | -0.077 | 0.349984 |
| 105926128 | LOC105926128 | metabotropic glutamate receptor 7-like | 1696.316 | 0.078 | 0.351148 |
| 105915552 | LOC105915552 | diacylglycerol kinase delta-like | 3833.656 | 0.138 | 0.352723 |
| 105918142 | rbm33 | RNA binding motif protein 33 | 2917.458 | 0.062 | 0.354904 |
| 105921585 | gramd1c | GRAM domain containing 1C | 67.555 | 0.132 | 0.358875 |
| 105940216 | itprip | inositol 1,4,5-trisphosphate receptor interacting protein | 78.968 | 0.138 | 0.358875 |
| 105916681 | LOC105916681 | integral membrane protein 2C-like | 4099.026 | 0.136 | 0.358875 |
| 105924148 | LOC105924148 | uncharacterized LOC105924148 | 412.563 | 0.111 | 0.358875 |
| 105926135 | LOC105926135 | prickle-like protein 2 | 3140.165 | 0.109 | 0.358875 |
| 105938174 | LOC105938174 | metal transporter CNNM4-like | 1068.297 | 0.110 | 0.358875 |
| 105915615 | camk2b | calcium/calmodulin dependent protein kinase II beta | 5500.911 | 0.094 | 0.361242 |
| 105915991 | LOC105915991 | guanine nucleotide-binding protein subunit alpha-14-like | 4.003 | 0.053 | 0.363025 |
| 105937875 | LOC105937875 | peptidyl-prolyl cis-trans isomerase FKBP1A-like | 4121.087 | 0.115 | 0.366327 |
| 105921784 | LOC105921784 | capZ-interacting protein-like | 101.515 | -0.139 | 0.369503 |
| 105939473 | LOC105939473 | beta-1,3-galactosyltransferase 2-like | 57.926 | 0.130 | 0.369867 |
| 105934387 | LOC105934387 | cytochrome c oxidase subunit 7A2, mitochondrial | 1871.910 | -0.102 | 0.370124 |
| 105933414 | sybu | syntabulin | 3847.436 | 0.070 | 0.371619 |
| 105932393 | btf3 | basic transcription factor 3 | 2554.223 | -0.071 | 0.371941 |
| 105937108 | slc16a10 | solute carrier family 16 member 10 | 413.499 | 0.117 | 0.372015 |
| 105922954 | LOC105922954 | dexamethasone-induced Ras-related protein 1-like | 253.644 | 0.134 | 0.373972 |
| 105938093 | LOC105938093 | EH domain-containing protein 4-like | 753.094 | -0.099 | 0.374356 |
| 105926622 | LOC105926622 | uncharacterized LOC105926622 | 5379.241 | 0.075 | 0.375274 |
| 105915896 | sacs | sacsin molecular chaperone | 5039.527 | 0.071 | 0.379009 |
| 105933004 | LOC105933004 | parvalbumin beta-like | 100.085 | 0.120 | 0.380536 |
| 105920316 | LOC105920316 | EF-hand domain-containing protein D1-like | 1176.553 | -0.085 | 0.380649 |
| 105916724 | LOC105916724 | myeloid-associated differentiation marker homolog | 52.251 | 0.114 | 0.381302 |
| 105938426 | bdh1 | 3-hydroxybutyrate dehydrogenase 1 | 1086.199 | -0.100 | 0.388191 |
| 105916643 | dmtn | dematin actin binding protein | 3417.736 | 0.091 | 0.388191 |
| 105937906 | nbr1 | NBR1, autophagy cargo receptor | 4430.180 | 0.053 | 0.388191 |
| 105925382 | gabra5 | gamma-aminobutyric acid type A receptor alpha5 subunit | 5216.158 | 0.106 | 0.389000 |
| 105932209 | LOC105932209 | sodium/calcium exchanger 1-like | 3771.833 | 0.136 | 0.390060 |
| 105916096 | LOC105916096 | sarcoplasmic/endoplasmic reticulum calcium ATPase 1 | 97.344 | 0.093 | 0.390663 |
| 105932309 | LOC105932309 | ataxin-1-like | 441.242 | 0.132 | 0.390663 |
| 105924544 | LOC105924544 | SMH class II histocompatibility antigen, beta-1 chain-like | 371.241 | -0.123 | 0.390701 |
| 105934315 | tax1bp1 | Tax1 binding protein 1 | 2511.163 | 0.069 | 0.394102 |
| 105920394 | LOC105920394 | sodium-coupled neutral amino acid transporter 3-like | 105.321 | 0.133 | 0.395992 |
| 105938872 | slc16a3 | solute carrier family 16 member 3 | 3257.548 | -0.072 | 0.396357 |
| 105919046 | zc3h4 | zinc finger CCCH-type containing 4 | 1256.476 | 0.082 | 0.396765 |
| 105921278 | dgkd | diacylglycerol kinase delta | 1683.407 | 0.070 | 0.397357 |
| 105930715 | LOC105930715 | tubulin alpha-1B chain-like | 10243.550 | -0.053 | 0.398502 |
| 105940396 | LOC105940396 | citron Rho-interacting kinase-like | 4526.614 | 0.136 | 0.399826 |

**Table S3.** Functional annotation biological processes of DEGs identified by RNA-seq. Gene Ontology (GO) enrichment analysis was performed by ToppFun web server (https://toppgene.cchmc.org/enrichment.jsp). Differentially expressed genes (q < 0.4) from DESEq2 analysis were entered into ToppGene. ToppFun selected analogous human symbols (e.g. cd83 became CD83) for about half of the 404 DEGs entered. The table is the Biological Processes portion.

| **ID** | **Name** | **pValue** | **FDR B&H** | **FDR B&Y** | **Bonferroni** | **Genes from Input** | **Genes in Annotation** |
| --- | --- | --- | --- | --- | --- | --- | --- |
| GO:0002520 | immune system development | 2.27E-07 | 4.36E-04 | 3.86E-03 | 8.81E-04 | 29 | 1127 |
| GO:1903706 | regulation of hemopoiesis | 3.46E-07 | 4.36E-04 | 3.86E-03 | 1.34E-03 | 19 | 538 |
| GO:0030097 | hemopoiesis | 3.47E-07 | 4.36E-04 | 3.86E-03 | 1.35E-03 | 27 | 1018 |
| GO:0010942 | positive regulation of cell death | 5.00E-07 | 4.36E-04 | 3.86E-03 | 1.94E-03 | 24 | 845 |
| GO:0043549 | regulation of kinase activity | 5.63E-07 | 4.36E-04 | 3.86E-03 | 2.18E-03 | 26 | 978 |
| GO:0001932 | regulation of protein phosphorylation | 7.70E-07 | 4.50E-04 | 3.98E-03 | 2.98E-03 | 35 | 1625 |
| GO:0048534 | hematopoietic or lymphoid organ development | 8.36E-07 | 4.50E-04 | 3.98E-03 | 3.24E-03 | 27 | 1065 |
| GO:0048167 | regulation of synaptic plasticity | 9.29E-07 | 4.50E-04 | 3.98E-03 | 3.60E-03 | 12 | 227 |
| GO:0034097 | response to cytokine | 1.10E-06 | 4.65E-04 | 4.11E-03 | 4.26E-03 | 30 | 1287 |
| GO:0010941 | regulation of cell death | 1.30E-06 | 4.65E-04 | 4.11E-03 | 5.05E-03 | 39 | 1969 |
| GO:0010770 | positive regulation of cell morphogenesis involved in differentiation | 1.42E-06 | 4.65E-04 | 4.11E-03 | 5.50E-03 | 11 | 195 |
| GO:0099537 | trans-synaptic signaling | 1.52E-06 | 4.65E-04 | 4.11E-03 | 5.90E-03 | 24 | 900 |
| GO:0045859 | regulation of protein kinase activity | 1.61E-06 | 4.65E-04 | 4.11E-03 | 6.25E-03 | 24 | 903 |
| GO:0019220 | regulation of phosphate metabolic process | 1.78E-06 | 4.65E-04 | 4.11E-03 | 6.89E-03 | 39 | 1994 |
| GO:0051174 | regulation of phosphorus metabolic process | 1.80E-06 | 4.65E-04 | 4.11E-03 | 6.98E-03 | 39 | 1995 |
| GO:0099536 | synaptic signaling | 1.95E-06 | 4.73E-04 | 4.18E-03 | 7.57E-03 | 24 | 913 |
| GO:0071345 | cellular response to cytokine stimulus | 2.14E-06 | 4.87E-04 | 4.31E-03 | 8.28E-03 | 28 | 1188 |
| GO:0042325 | regulation of phosphorylation | 2.30E-06 | 4.95E-04 | 4.38E-03 | 8.92E-03 | 36 | 1781 |
| GO:0051338 | regulation of transferase activity | 4.10E-06 | 7.76E-04 | 6.86E-03 | 1.59E-02 | 26 | 1089 |
| GO:0010769 | regulation of cell morphogenesis involved in differentiation | 4.35E-06 | 7.76E-04 | 6.86E-03 | 1.69E-02 | 14 | 360 |
| GO:0098916 | anterograde trans-synaptic signaling | 4.40E-06 | 7.76E-04 | 6.86E-03 | 1.71E-02 | 23 | 891 |
| GO:0007268 | chemical synaptic transmission | 4.40E-06 | 7.76E-04 | 6.86E-03 | 1.71E-02 | 23 | 891 |
| GO:0051094 | positive regulation of developmental process | 6.64E-06 | 1.10E-03 | 9.76E-03 | 2.58E-02 | 33 | 1633 |
| GO:0001775 | cell activation | 6.83E-06 | 1.10E-03 | 9.76E-03 | 2.65E-02 | 32 | 1559 |
| GO:0050773 | regulation of dendrite development | 8.36E-06 | 1.30E-03 | 1.15E-02 | 3.24E-02 | 10 | 191 |
| GO:0022604 | regulation of cell morphogenesis | 8.94E-06 | 1.33E-03 | 1.18E-02 | 3.47E-02 | 17 | 551 |
| GO:0016358 | dendrite development | 1.33E-05 | 1.84E-03 | 1.63E-02 | 5.14E-02 | 12 | 294 |
| GO:0043085 | positive regulation of catalytic activity | 1.33E-05 | 1.84E-03 | 1.63E-02 | 5.16E-02 | 32 | 1611 |
| GO:0050890 | cognition | 1.94E-05 | 2.59E-03 | 2.29E-02 | 7.50E-02 | 13 | 357 |
| GO:0060284 | regulation of cell development | 2.44E-05 | 3.15E-03 | 2.78E-02 | 9.45E-02 | 25 | 1133 |
| GO:0071407 | cellular response to organic cyclic compound | 2.53E-05 | 3.17E-03 | 2.80E-02 | 9.82E-02 | 18 | 660 |
| GO:0050804 | modulation of chemical synaptic transmission | 2.66E-05 | 3.18E-03 | 2.81E-02 | 1.03E-01 | 16 | 539 |
| GO:0099177 | regulation of trans-synaptic signaling | 2.72E-05 | 3.18E-03 | 2.81E-02 | 1.05E-01 | 16 | 540 |
| GO:0007611 | learning or memory | 2.79E-05 | 3.18E-03 | 2.81E-02 | 1.08E-01 | 12 | 317 |
| GO:0010976 | positive regulation of neuron projection development | 2.89E-05 | 3.20E-03 | 2.83E-02 | 1.12E-01 | 13 | 371 |
| GO:0010975 | regulation of neuron projection development | 3.56E-05 | 3.83E-03 | 3.39E-02 | 1.38E-01 | 17 | 614 |
| GO:0042981 | regulation of apoptotic process | 3.82E-05 | 4.00E-03 | 3.54E-02 | 1.48E-01 | 33 | 1779 |
| GO:0050808 | synapse organization | 4.05E-05 | 4.13E-03 | 3.65E-02 | 1.57E-01 | 15 | 498 |
| GO:0030217 | T cell differentiation | 4.33E-05 | 4.18E-03 | 3.69E-02 | 1.68E-01 | 11 | 280 |
| GO:0120035 | regulation of plasma membrane bounded cell projection organization | 4.48E-05 | 4.18E-03 | 3.69E-02 | 1.74E-01 | 20 | 822 |
| GO:0050865 | regulation of cell activation | 4.52E-05 | 4.18E-03 | 3.69E-02 | 1.75E-01 | 18 | 690 |
| GO:0045597 | positive regulation of cell differentiation | 4.56E-05 | 4.18E-03 | 3.69E-02 | 1.77E-01 | 25 | 1177 |
| GO:0048639 | positive regulation of developmental growth | 4.63E-05 | 4.18E-03 | 3.69E-02 | 1.80E-01 | 10 | 233 |
| GO:0051247 | positive regulation of protein metabolic process | 4.87E-05 | 4.29E-03 | 3.79E-02 | 1.89E-01 | 34 | 1882 |
| GO:1900006 | positive regulation of dendrite development | 5.10E-05 | 4.39E-03 | 3.88E-02 | 1.98E-01 | 7 | 108 |
| GO:0031344 | regulation of cell projection organization | 5.29E-05 | 4.46E-03 | 3.94E-02 | 2.05E-01 | 20 | 832 |
| GO:0043067 | regulation of programmed cell death | 5.43E-05 | 4.48E-03 | 3.96E-02 | 2.11E-01 | 33 | 1811 |
| GO:0043065 | positive regulation of apoptotic process | 6.02E-05 | 4.87E-03 | 4.30E-02 | 2.34E-01 | 19 | 772 |
| GO:0030098 | lymphocyte differentiation | 6.91E-05 | 5.36E-03 | 4.74E-02 | 2.68E-01 | 13 | 404 |
| GO:0043068 | positive regulation of programmed cell death | 7.02E-05 | 5.36E-03 | 4.74E-02 | 2.72E-01 | 19 | 781 |

**Table S4.** Cycle threshold (Ct) values from qPCR. Each value represents the average value from reactions performed in triplicate. Housekeeper gene Ct scores were retested when reagents for an assay were obtained from different kits. Sample sizes were from eight to ten individuals.

|  | **No Injury** | | | **Intact** | | | **Lesioned** | | |
| --- | --- | --- | --- | --- | --- | --- | --- | --- | --- |
|  | Housekeeper | Gene of interest | | Housekeeper | Gene of interest | | Housekeeper | Gene of interest | |
| Individual | *ef1a1* | *pim2-like* | *syndecan-4-like* | *ef1a1* | *pim2-like* | *syndecan-4-like* | *ef1a1* | *pim2-like* | *syndecan-4-like* |
| 1 | 21.24 | 26.15 | 26.66 | 21.24 | 25.59 | 26.48 | 21.39 | 24.29 | 25.26 |
| 2 | 21.65 | 27.91 | 26.56 | 21.33 | 25.70 | 25.73 | 21.46 | 24.68 | 25.48 |
| 3 | 21.55 | 26.64 | 26.99 | 21.42 | 23.87 | 25.56 | 21.85 | 22.86 | 25.07 |
| 4 | 21.31 | 25.89 | 26.08 | 21.19 | 25.29 | 26.59 | 21.03 | 23.78 | 25.31 |
| 5 | 20.65 | 25.25 | 26.54 | 21.38 | 25.91 | 25.83 | 21.37 | 24.10 | 25.24 |
| 6 | 22.18 | 26.17 | 27.07 | 21.36 | 26.60 | 26.19 | 21.42 | 25.17 | 26.35 |
| 7 | 20.79 | 25.91 | 26.27 | 21.66 | 26.26 | 26.02 | 22.13 | 23.91 | 25.12 |
| 8 | 21.67 | 26.48 | 26.84 | 21.25 | 26.74 | 26.87 | 22.46 | 24.64 | 26.54 |
| 9 | 21.11 | 27.02 | 26.41 | 21.18 | 24.52 | 26.25 | 21.33 | 23.32 | 25.69 |
| 10 | 21.21 | 27.62 | 26.63 | 21.78 | 24.57 | 25.67 | 21.44 | 23.14 | 25.06 |
| Individual | *ef1a1* | *cd83* | | *ef1a1* | *cd83* | | *ef1a1* | *cd83* | |
| 1 | 21.71 | 28.51 | | 21.60 | 27.73 | | 21.64 | 24.11 | |
| 2 | 21.83 | 28.35 | | 21.70 | 26.04 | | 21.53 | 25.28 | |
| 3 | 21.72 | 30.57 | | 21.49 | 28.66 | | 21.01 | 25.90 | |
| 4 | 21.40 | 27.61 | | 21.35 | 26.96 | | 21.21 | 25.34 | |
| 5 | 20.99 | 25.13 | | 21.74 | 26.08 | | 21.87 | 25.26 | |
| 6 | 22.02 | 28.66 | | 21.94 | 26.42 | | 21.55 | 26.08 | |
| 7 | 21.12 | 25.90 | | 22.07 | 25.46 | | 22.19 | 24.95 | |
| 8 | 20.95 | 26.44 | | 20.41 | 25.15 | | 21.09 | 23.97 | |
| 9 | 21.04 | 27.29 | | 21.62 | 26.75 | | 21.45 | 27.04 | |
| 10 | 21.72 | 29.10 | | 21.46 | 25.03 | | 21.68 | 24.83 | |
| Individual | *ef1a1* | *igf-1* | | *ef1a1* | *igf-1* | | *ef1a1* | *igf-1* | |
| 1 | 21.23 | 26.40 | | 21.47 | 26.54 | | 21.62 | 26.90 | |
| 2 | 21.43 | 27.39 | | 21.60 | 26.71 | | 21.67 | 26.97 | |
| 3 | 21.70 | 26.80 | | 21.30 | 26.56 | | 21.42 | 26.64 | |
| 4 | 21.51 | 26.25 | | 21.69 | 26.78 | | 21.83 | 26.91 | |
| 5 | 21.37 | 26.98 | | 21.95 | 26.94 | | 22.46 | 26.66 | |
| 6 | 21.73 | 26.94 | | 21.51 | 26.78 | | 21.62 | 27.00 | |
| 7 | 21.97 | 27.12 | | 21.58 | 26.26 | | 21.73 | 26.57 | |
| 8 | 21.82 | 26.72 | | 21.39 | 26.78 | | 21.98 | 26.84 | |
